# Effects of continuous renal replacement therapy on intestinal mucosal barrier function during extracorporeal membrane oxygenation in a porcine model

**DOI:** 10.1186/1749-8090-9-72

**Published:** 2014-04-23

**Authors:** Changsheng He, Shuofei Yang, Wenkui Yu, Qiyi Chen, Juanhong Shen, Yimin Hu, Jialiang Shi, Xingjiang Wu, Jieshou Li, Ning Li

**Affiliations:** 1 Department of General Surgery, Jinling hospital, Medical School of Nanjing University, Nanjing 210002, P.R. China; 2 Clinical School of Second Military Medical University, Nanjing 210002, P.R. China; 3 Clinical School of Nanjing University, Nanjing 210002, P.R. China

**Keywords:** Extracorporeal membrane oxygenation (ECMO), Continuous renal replacement treatment (CRRT), Systemic inflammatory response syndrome (SIRS), Intestinal mucosal barrier function

## Abstract

**Backgrounds:**

Extracorporeal membrane oxygenation (ECMO) has been recommended for treatment of acute, potentially reversible, life-threatening respiratory failure unresponsive to conventional therapy. Intestinal mucosal barrier dysfunction is one of the most critical pathophysiological disorders during ECMO. This study aimed to determine whether combination with CRRT could alleviate damage of intestinal mucosal barrier function during VV ECMO in a porcine model.

**Methods:**

Twenty-four piglets were randomly divided into control(C), sham(S), ECMO(E) and ECMO + CRRT(EC) group. The animals were treated with ECMO or ECMO + CRRT for 24 hours. After the experiments, piglets were sacrificed. Jejunum, ileum and colon were harvested for morphologic examination of mucosal injury and ultrastructural distortion. Histological scoring was assessed according to Chiu’s scoring standard. Blood samples were taken from the animals at -1, 2, 6, 12 and 24 h during experiment. Blood, liver, spleen, kidney and mesenteric lymphnode were collected for bacterial culture. Serum concentrations of diamine oxidase (DAO) and intestinal fatty acid binding protein (I-FABP) were tested as markers to assess intestinal epithelial function and permeability. DAO levels were determined by spectrophotometry and I-FABP levels by enzyme linked immunosorbent assay.

**Results:**

Microscopy findings showed that ECMO-induced intestinal microvillus shedding and edema, morphological distortion of tight junction between intestinal mucous epithelium and loose cell-cell junctions were significantly improved with combination of CRRT. No significance was detected on positive rate of serum bacterial culture. The elevated colonies of bacterial culture in liver and mesenteric lymphnode in E group reduced significantly in EC group (p < 0.05). Compared with E group, EC group showed significantly decreased level of serum DAO and I-FABP (p < 0.05).

**Conclusions:**

CRRT can alleviate the intestinal mucosal dysfunction and bacterial translocation during VV ECMO, which may extenuate the ECMO-associated SIRS and raise the clinical effect and safety.

## Background

Extracorporeal membrane oxygenation (ECMO) has been considered as an effective means of treatment to unresponsive pulmonary hypertension, respiratory failure, sepsis, and emergency temporary cardiac support for almost 60 years [1,2]. With superiority for moribund patients, venovenous extracorporeal membrane oxygenation (VV ECMO) is recommended to support patients with severe but potentially reversible respiratory failure refractory to conventional therapy [3-6]. The encouraging results in the CESAR trial and remarkable effect of ECMO in 2009 H1N1 and 2013 H7N9 pandemichas brought it into the spotlight worldwide [7-10]. However, direct circulation of blood across synthetic surfaces escalates a pro-inflammatory response, further exacerbating a disease process that is already associated with the activation of the inflammatory cascade [11]. With much wider range of use, concerns remain about the near-universal occurrence of systemic inflammatory response syndrome (SIRS) during ECMO associated with considerable morbidity [12-17]. Intestine is the central part of systemic SIRS and a motor of multiple organ dysfunction syndrome (MODS) [18]. Rapid rise of plasma concentrations of inflammatory cytokines during ECMO-related SIRS is due to the release of pre-formed stores in the intestine [19]. Breakdown of mucosal barrier during ECMO increases intestinal permeability and allows translocation of intraluminal bacteria across the mucosa leading to distant organ injury [15]. Strategies to protect gut barrier function and thereby prevent bacterial translocation during ECMO merit comprehensive and further explorations.

Continuous renal replacement therapy (CRRT) shows remarkable advantages in eliminating inflammatory mediators, dampening inflammatory response, preserving organ function and maintaining optimal fluid status during ECMO with well clinical tolerance in a hemodynamically unstable condition [20-22]. ECMO-associated organ lesion of myocardium and kidney was proven to be extenuated with CRRT [23]. As far as we know, this is the first study designed to investigate whether CRRT could alleviate the gut mucosal dysfunction and intestinal epithelial barrier loss in a swine model so as to provide an approach to improve the clinical effect and relieve the risk of VV ECMO therapy.

## Methods

This study was approved by the Institutional Animal Care and Use Committee of Jingling Hospital in Nanjing, Jiangsu, China. Experiments were performed according to the National Institutes of Health Guidelines on the use of laboratory animals.

### Experimental protocol

The 24 piglets weight of 27.46 ± 4.45 Kg (25-32 Kg) of either sex were randomly allocated to 4 groups: control group (C group), sham group (S group, to verify whether the required ECMO operative procedure affected the results), VV-ECMO group (E group), VV-ECMO combined with CRRT group (EC group). All piglets were fasted for 24 h before experiments. Each animal received lactated Ringer’s solution at a rate of 3 mL*kg^−1^*h^−1^ and bolus fluid was provided as required to maintain MAP above 60 mmHg. Temperature, serum pH, glucose, and ionized calcium concentration were monitored and maintained normally. All animals were monitored for 24 h. After heparin (150 U/kg IV) was given as a bolus to the sham, ECMO and ECMO + CRRT groups, placement of cannula was performed and confirmed by ultrasonograph. The control group got no operation and treatment. In sham group, the vascular venous cannula was occluded at 0 hour. In ECMO group, ECMO was instituted with cannulae insertion. In ECMO + CRRT group, ECMO and CRRT were initiated at the same time upon cannulae insertion. The blood and tissue samples were collected 1 h before and 2 h, 6 h, 12 h and 24 h after experiment. At the end of experiments, piglets were euthanized with a bolus injection of potassium chloride (40 mL, 0.1 g/mL).

### Surgical procedure

Anesthesia was induced with ketamine (20 mg/kg), diazepam (8 mg/kg), and atropine (0.1 mg/kg) intramuscularly and then maintained with ketamine (10–20 mg*kg^−1^*h^−1^) and diazepam (8 mg*kg^−1^*h^−1^) intravenously. The animals were intubated through a cervical tracheotomy and then mechanical ventilation (TBird VELA, USA) was established using volume-controlled mode with FiO2 of 21% and a positive end-expiratory pressure set at 5 mmHg throughout the experimental period. Tidal volumes were adjusted to 5-8 mL/kg. Respiratory rate was set at 15/min. Internal jugular vein and femoral vein catheters were placed for intravenous access, sample collection and connected to a medical monitor (Model90207, Spacelabs, USA).

### VV-ECMO proceduce

After a 150 U/kg intravenous bolus of heparin, ECMO cannulae (15-Fr, Medtronic, USA) were placed in the superior vena cava through the internal jugular vein and inferior vena cava through the femoral vein. Placement of cannulae was confirmed under guidance of ultrasonograph. Heparin was continuously infused to keep the activated clotting time (ACT) at 180 to 220 s during the process of ECMO. The ECMO system consisted of a membrane oxygenator and tubing (Quadrox PLS, Maquet, Germany), a centrifugal pump (Rotaflow Console, Maquet, Germany), and a heat exchange maintaining temperature at 37°C (Heater–Cooler Unit HCU 30, Maquet, Germany). The circuit was primed with 500 mL hydroxyethyl starch 130/0.4 and 200-300 mL Ringer’s lactate. Blood in circuit was drained from the femoral vein cannulae and infused into the internal jugular vein cannulae at a flow rate of 50 mL*kg^−1^*min^−1^. Sweep gas was 100% oxygen at a flow rate equal to the blood flow rate.

### CRRT setting

CRRT was performed in a pre-dilution mode using a polysulfone membrane (1.4 m2 AV 600 s, ultraflux, Germany) connected to a continuous blood pump (Baxter BM 25, Baxter SAN Germany). CRRT was performed with zero-balanced continuous venovenous hemofiltration (CVVH) at a blood flow of 160-180 mL/min and an ultrafiltration rate of 35 mL*kg^−1^*h^−1^. A lactate-buffered replacement fluid (deploy Jingling prescription, China) was administered in a post-dilutional fashion.

### Connecting manner

The inlet (arterial) line of the CRRT circuit was connected after oxygenator by a three-way tap and the outlet (venous) line was connected to the circuit at another tap on infusing cannula. The filters were unchanged during 24 hours each time.

### Histopathological examination

1. Histology examination and score

Tissue samples from the jejunum, ileum and colon were fixed and dehydrated with 10% formalin, embedded in paraffin, cutted into sections, stained with hematoxylin and eosin (H and E staining), and observed in 100-times by an optical microscopy (CX41, Olympus, Tokyo, Japan). Four distinct sections of each sample are observed and scored according to Chiu’s scoring standard by two independent pathologist blinded to the grouping [24].

2. Ultrastructure detection

Fresh intestinal tissues from the jejunum, ileum and colon were flushed with phosphate-buffered saline, cut into pieces (1*1*1 mm), fixed with 2.5% cold glutaraldehyde for 2 h, flushed with phosphate-buffered saline again, fixed with 1% perosmic acid, and dehydrated with acetone. Ultrathin sections were placed on 200-mesh copper grids and double stained with 4% uranyl acetate and 0.2% lead citrate. Sections were examined under transmission electron microscopy (TEM, JEM-1200, Hitachi, Tokyo, Japan). Ultrastructure of tight junction was observed in 10,000-times magnification. The histology and ultrastructure were examined by two independent pathologist blinded to the grouping.

### Measurement of intestinal barrier function

1. Bacterial translocation

a) Blood bacterial culture: blood samples were placed in agarose-gel plates to aerobic and anaerobic bacterial culture for 24 h for bacterial identification.

b) Tissue bacterial culture: after animals were euthanized, the liver, spleen, kidney and mesenteric lymphnode were collected by stringent aseptic operation for tissue bacterial culture at 37°C for 24 h (measured by clonal formation unit/gram, CFU/g).

2. Concentrations of Diamine Oxidase (DAO) and intestinal fatty acid binding protein (I-FABP)

Concentrations of DAO and I-FABP were used as the markers for the assessment of intestinal epithelial function. Serum and filtrate DAO levels were determined using a spectrophotometry kit (Nanjing Jiancheng Bioengineering Institute,China). Serum and filtrate I-FABP level were tested by enzyme linked immunosorbent assay (ELISA) kit (Wuhan EIAab Science Co., Ltd, China). Spectrophotometry and ELISA was performed according to the manufacturer’s instructions.

### Statistical analysis

Continuous variables were defined as means ± standard deviation if they were normally distributed,Colonies of tissue bacterial culture were computed by log10. Comparisons between groups for pathological score, colonies of tissue bacterial culture, DAO and I-FABP levels in filtrate were performed by using one-way analysis of variance (ANOVA). In case of statistical significance, post hoc comparisons were made by unpaired samples t-test. Fisher’s exact test was used in blood culture positive rate. Repeated-measures ANOVA were used to analyze DAO and I-FABP variables over time between four groups followed by Bonferroni’s post hoc testing. Statistical comparisons among the groups were performed using two-way ANOVA, followed by unpaired t-tests with Bonferroni’s correction. Statistical significance was accepted for a P-value of 0.05. Statistical analysis was performed using the SPSS statistical package (version17.0; SPSS Inc., Chicago, IL, USA).

## Results

No significant differences were detected in body weight and other characteristics among groups.

### Histology and ultrastructure examination

**Figure 1:**
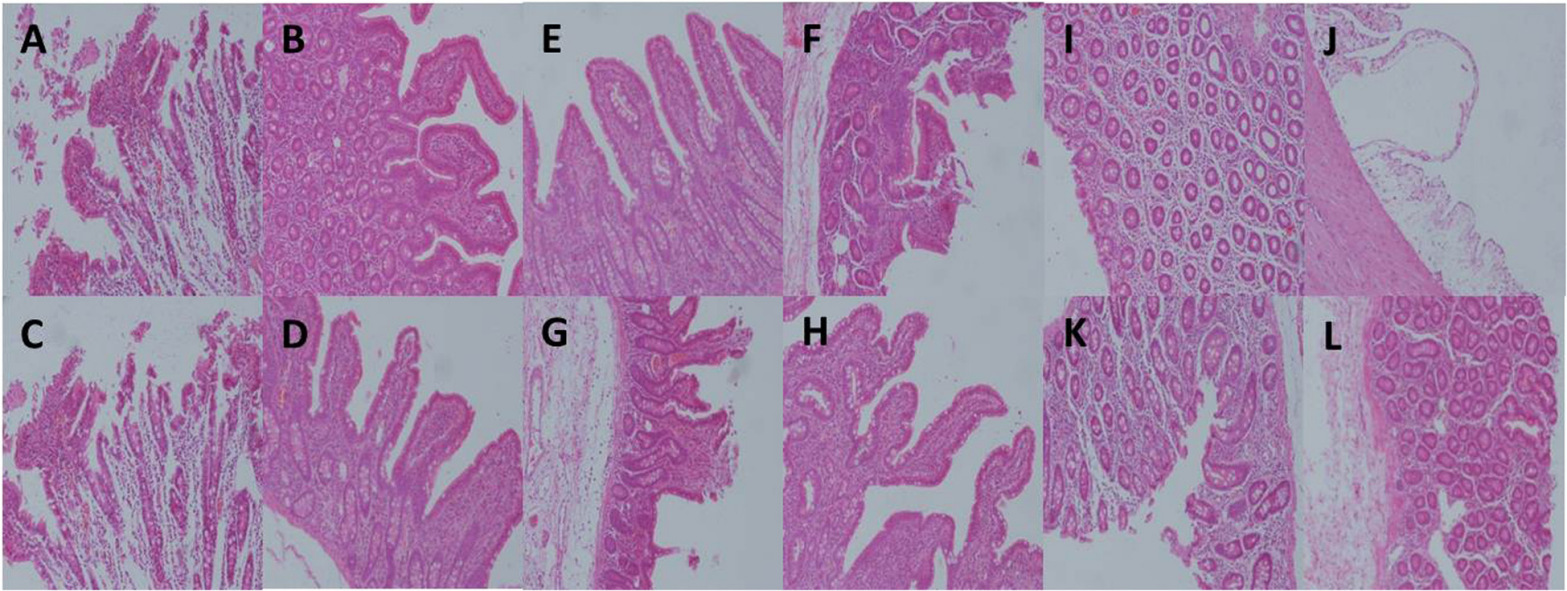
Histology examination of jejunal, ileal and colonic mucosa with H and E staining under optical microscopy (magnification*100). A-D: jejunal mucosa of C, S, E, EC group; **E-H**: ileal mucosa of C, S, E, EC group; **I-L**: colonic mucosa of C, S, E, EC group. Normal mucosa was viewed in C group. Mucosal structure distortion, villous collapse and edema, and epithelial shedding were observed in S and E groups. These damages were significantly attenuated in EC group.

**Figure 2:**
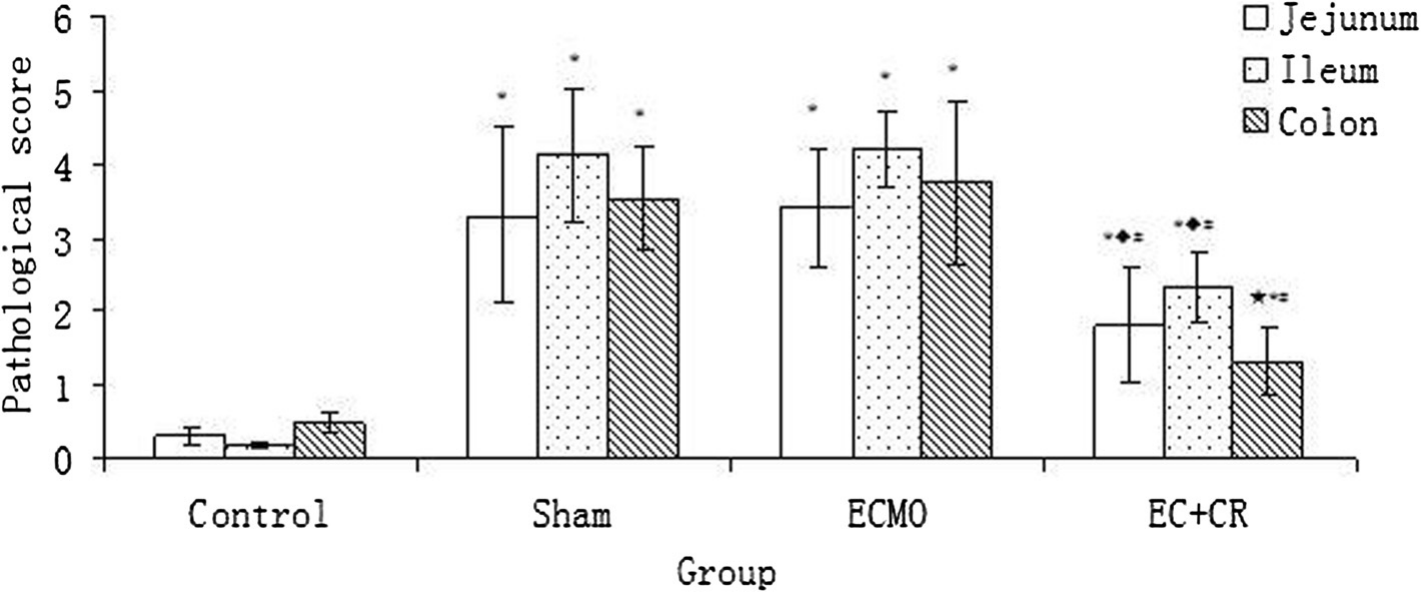
**Pathological score of jejunal, ileal and colonic mucosa injury.** Socres of S and E group were markedly higher than C group (P < 0.01). Score of EC group was significantly lower than S and E group, but higher than C group (P < 0.01). (Compared to control group, ^*^P < 0.01, ^★^P < 0.05; compared to S group, ^◆^P < 0.01; compared to E group, ^#^P < 0.01).

In histology examination, jejunal and ileal mucosa was normal in C group. Jejunal and ileal mucosal structure distortion, villous collapse and edema, and epithelial shedding were observed in S and E groups. These damages were attenuated in EC group with slight mucosal injury and villi shedding (Figure 1A-H). Normal morphology of colonic mucosa was demolished in E group and slight structural disorder with epithelial atrophy was found in S group. Mucosal damages were relieved back to smooth and integral in EC group similar to C group (Figure 1I-L). Significant difference of pathologic scores was detected among four groups (P < 0.01). Socres of S and E group were markedly higher than C group, disclosing the impairment on intestinal mucosa by invasive manipulation and ECMO therapy (P < 0.01). Score was slightly elevated in E group when compared with S group. Score of EC group was significantly lower than S and E group, but higher than C group (P < 0.01). It revealed that CRRT could alleviate the ECMO-related morphological distortion of intestial mucosa (Figure 2).

**Figure 3:**
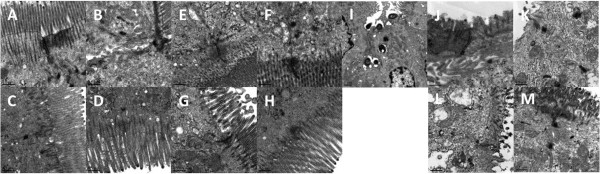
Ultrastructural detection of jejunal, ileal and colonic mucosa under transmission electron microscopy (magnification*10,000). A-D: jejunal mucosa of C, S, E, EC group; **E-H**: ileal mucosa of C, S, E, EC group; **I**: bacterial invasion into ileal mucosa; **J-M**: colonic mucosa of C, S, E, EC group. The morphological distortion of tight junction between intestinal mucous epithelium became blurred with loose cell-cell gaps, disappearance of desmosomes and mitochondrial swelling in E group were significantly improved in EC group.

Under TEM, almost the same in jejunum, ileum and colon, structural integrity and solid connection of epithelial tight junction and desmosome with smooth microvillus were indicated in C group. Loose epithelial tight junction and sparse villi were found in S group. The morphological distortion of tight junction between intestinal mucous epithelium became blurred with loose cell-cell gaps, disappearance of desmosomes and mitochondrial swelling in E group. All damages were significantly improved in EC group with solid epithelial tight junction, dense desmosome and tidy villi (Figure 3A-H, J-M). Bacterial invasion into ileal mucosa was clearly observed suggesting a significant increase of intestinal mucosal barrier permeability (Figure 3I).

### Bacterial translocation

**Figure 4:**
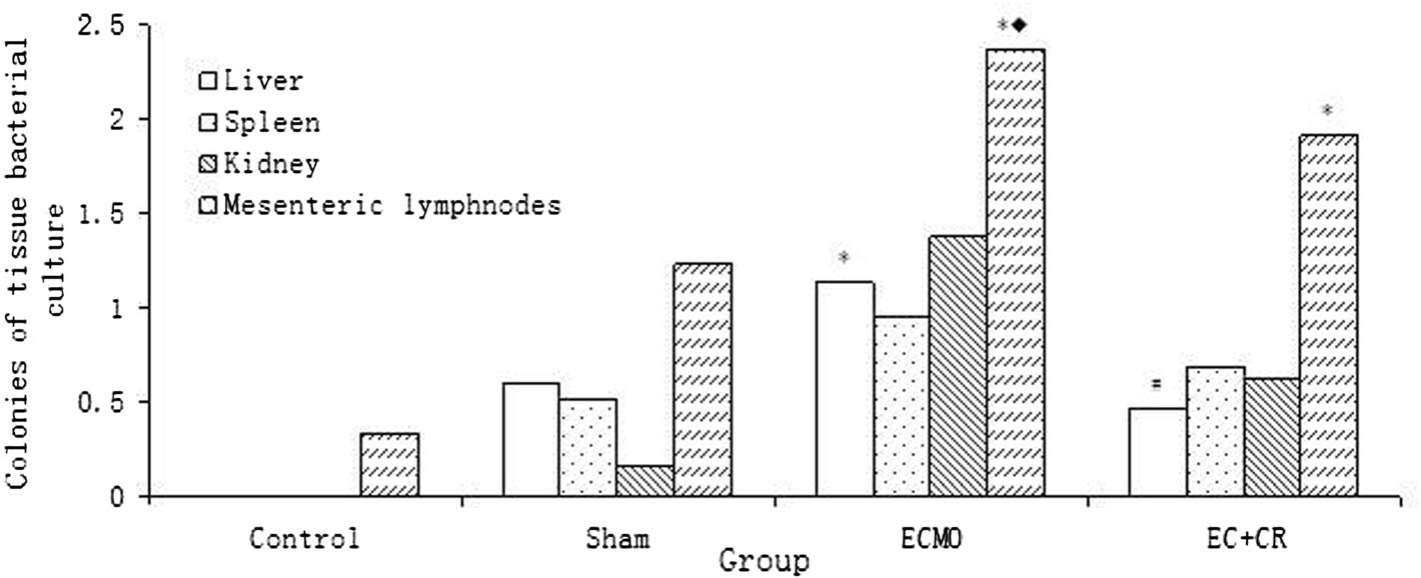
**Colonies of bacterial culture in liver, spleen, kidney and mesenteric lymphnode.** VV ECMO therapy (E group) induced a significant increase of colonies of bacterial culture in liver and mesenteric lymphnode than C group (P < 0.01), which was attenuated significantly in liver (P < 0.01) and partially in mesenteric lymphnode after combination with CRRT therapy (EC group). (Compared to control group, *P < 0.01; compared to S group, ◆P < 0.05; compared to E group, #P < 0.05).

Blood bacterial culture was negative in all groups. At 24 h after experiment, positive rate of blood bacterial culture was highest in E group (33.33%), lower in S and EC group (16.67%) and none in C group. No statistical difference was detected by Fisher’s exact test (Table 1). There was significant difference in bacterial culture of liver and mesenteric lymphnode among four groups (P < 0.05), but none in kidney and spleen (P > 0.05). VV ECMO therapy (E group) induced a significant increase of colonies of liver and mesenteric lymphnode bacterial culture than C group (P < 0.01) which was attenuated significantly in liver (P < 0.01) and partially in mesenteric lymphnode after combination with CRRT therapy (EC group). Colonies of mesenteric lymphnode bacterial culture rose notably in E group (vs S group, P < 0.05) and EC group (vs C group, P < 0.01). Colonies of liver bacterial culture increased mildly in E group (vs S group, P = 0.06) and EC group (vs C group, P = 0.15) (Figure 4).

**Table 1:** Serum bacterial culture

**Group**	**Before experiment (%)**	**24 h after experiment (%)**
Control	0/6 (0)	0/6 (0)
Sham	0/6 (0)	1/6 (16.66)
ECMO	0/6 (0)	2/6 (33.33)
EC + CR	0/6 (0)	1/6 (16.66)

### Injury of intestinal epithelial barrier

**Figure 5:**
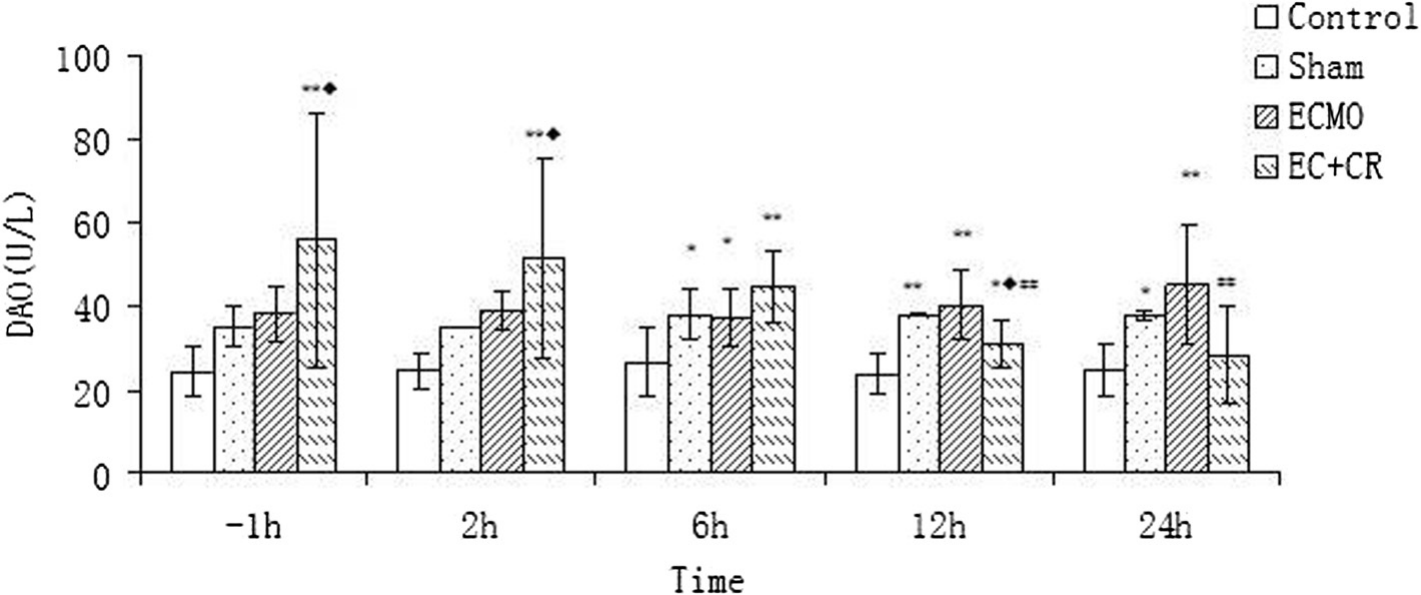
**Serum DAO levels in 4 groups.** Serum DAO levels did not change significantly over time (P > 0.05). Compared with notable increase of serum DAO level in E group, it was significantly lower after CRRT for 12 h (P < 0.01). (Compared to control group, *P < 0.05, **P < 0.01; compared to S group, ◆P < 0.05, ◆◆P < 0.01; compared to E group, #P < 0.05, ##P < 0.01).

**Figure 6:**
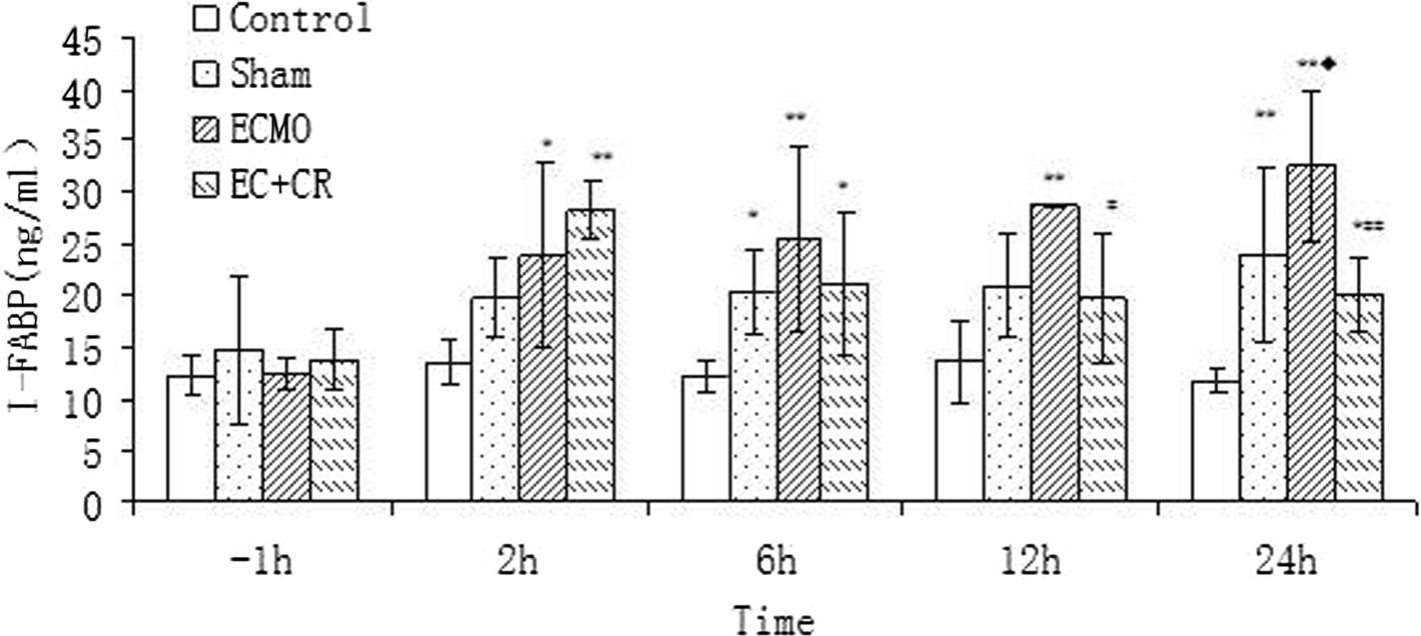
**Serum I-FABP levels in 4 groups.** Serum I-FABP levels did not change significantly over time (P > 0.05). Compared with high serum I-FABP level in E group, it showed significant decrease after CRRT for 12 h (P < 0.05). (Compared to control group, *P < 0.05, **P < 0.01; compared to S group, ◆P < 0.05, ◆◆P < 0.01; compared to E group, #P < 0.05, ##P < 0.01).

**Figure 7:**
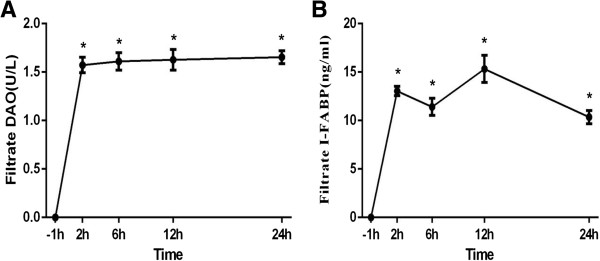
**Filtrate DAO and I-FABP level over time. A**: Filtrate DAO and I-FABP level over time; **B**: Filtrate DAO and I-FABP level over time. The filtrate DAO and I-FABP level remained relatively constant throughout the experiment period. (Compared to -1 h, *P < 0.05).

Serum DAO and I-FABP levels did not change over time significantly among 4 groups (P > 0.05). But they differed significantly between groups at the same time point (P < 0.05). Compared with constant low level in C group, S and E group demonstrated notable increase of serum DAO and I-FABP level (P < 0.05, P < 0.01). In EC group, despite initial high level, after 2 h of CRRT serum DAO and I-FABP levels decreased gradually to same level as C group at 24 h, significantly lower than E group (P < 0.01) (Figures 5 and 6). The filtrate DAO and I-FABP levels remained relatively constant throughout the study period, which demonstrated that decreased serum DAO and I-FABP levels in EC group was not due to clearance of CRRT (Figure 7A, B). All the results suggested that the VV ECMO-induced intestinal mucosal barrier dysfunction could be relieved notably with combination of CRRT.

## Discussion

To date, our findings are the first in vivo animal experiment in an VV ECMO model to demonstrate the protective effect of CRRT on intestinal mucosal barrier dysfunction secondary to ECMO-induced SIRS. During ECMO, huge disparity between the patient’s circulating blood volume and circuit volumes, often 200–300% greater than patient’s circulating blood volume, increases the activation of various blood components upon long exposure to the artificial material surface of the ECMO circuit leading to changes of blood constituent and SIRS [15,25]. This inflammatory response usually manifests within the first few hours of ECMO with hypotension, decreased urine output and lung compliance, anasarca and liver dysfunction [26]. These changes often persist for several days with delayed recovery and prolonged ECMO therapy. A reciprocal causation is found between intestinal mucosal barrier dysfunction and inflammatory cytokine storm. Rapid rise of inflammatory cytokines during ECMO-related SIRS was due to the release of pre-formed stores in the intestine [19]. On the other side, loss of intestinal epithelial barrier secondary to ECMO-indeced SIRS has been demonstrated in previous study and similar changes were detected in this study as well [15]. In the present study, bacterial translocation was detected after the initiation of ECMO. Similarly, Hirthler et al. [27] found increased plasma LPS levels in neonates after initiation of ECMO for 36 h. Bacterial translocation due to the increase of gut mucosal permeability in human neonates receiving ECMO has also been noted by Piena et al. [28]. Bacterial products play an important role in initiating and amplifying all the major inflammatory pathways involved in ECMO-related SIRS [29]. Since the early development of intestinal mucosal barrier dysfunction and bacterial translocation on initiation of ECMO, it is more likely that intestinal epithelial barrier injury acts as a primary initiator or amplifier of ECMO-related SIRS rather than an epiphenomenon reflecting loss of mucosal integrity.

Concentrations of DAO and I-FABP were used as quantitative method to assess the intestinal epithelial function in this study. DAO is a kind of highly-active enzyme weight of 250 kD existed only in intestinal epithelium. About 95% of DAO locates in the intestinal villi of human and other mammals. It will transfer into peripheral blood in a stable state upon intestinal mucosal barrier dysfunction. I-FABP is low-molecular-weight protein specifically located in epithelial cells of small bowel mucosa which is rapidly released into the circulation after injury of intestinal mucosal barrier due to mesenteric ischemia and bowel necrosis [30]. DAO and I-FABP are excellent early marker for evaluating the severity of acute intestinal mucosa injury [31]. In 2004, Nobuo Tsunooka et al. [32] evaluated bacterial translocation secondary to small intestinal mucosal ischemia during cardiopulmonary bypass by serum level of DAO and peptidoglycan. DAO was used as an index of beneficial effect of continuous blood purification (CBP) on gut barrier dysfunction in another study [33]. In our study, after 2 h of CRRT, the intestine mucosal dysfunction indicated by significantly elevated serum level of DAO and I-FABP in E group declined and returned to almost the same level with C group at 24 h. Since the DAO and I-FABP level in filtrate did not elevate as CRRT continued, perhaps reaching a saturation limit, decrease of serum DAO and I-FABP level were due to improvement of intestine mucosal function instead of the clean-up effect.

Because of the inherent characteristics of ECMO circuit to activate inflammatory pathways, elucidation of inflammatory pathways by CRRT is a critical step in the development of effective strategy to ECMO-related SIRS [34]. But the definitive mechanism of the protective effects of CRRT to ECMO-induced injury of intestinal epithelial barrier is not yet clear. Intestinal mucosal edema with epithelial swelling, cell-cell gap expanding, tight junction and desmosome loosening was clearly observed during ECMO. Optimal fluid status maintained by CRRT can effectively reduce the high permeability secondary to intestinal mucosal edema. Additionally, direct cytotoxic effects of massive inflammatory cells or cytokines and their role in the development of reactive oxygen species (ROS) and mitochondrial dysfunction may be another main reason for ECMO-associated intestinal epithelial barrier injury [23]. CRRT operates with the ability of inflammatory mediator elimination via several ways, including absorption, diffusion, and convection, dampening inflammatory response to preserve organ function [35,36]. Moreover, CRRT could ameliorate the gut barrier dysfunction by way of attenuating breakdown and reorganization of tight junction protein, such as occludin and zonula occludens-1, with increase of the inflammation-induced nitric oxide synthase (iNOS) mRNA levels and nitric oxide (NO) production which are implicated as factors contributing to cytoskeletal instability and injury [33,37]. Phosphodiesterase inhibition is another possible mechanism of CRRT-mediated attenuation of gut barrier injury [38,39]. Further studies are warrented to identify specific mediators and pathways of the protective effects of CRRT on intestinal epithelial barrier during ECMO.

A high prevalence of acute renal failure requiring renal replacement therapy in patients treated with ECMO has been observed [40,41]. Expanding CRRT to non-renal indications, such as cardopulmonary bypass and sepsis, based on elimination of inflammatory mediators has been suggested [42]. Our study provides an additional evidence to initiate CRRT during VV ECMO before renal dysfunction. However, there are some underlying matters and limitations of this study. Similar to other studies, our experimental timeline spanned only 24 h and may limit its applicability in clinical practice where ECMO therapy lasts for days to weeks. The effect of longterm consequences of this strategy on enterogenic infection, general intestinal function, hemodynamic alteration and oxygen utilization warrant further study. The differences between patient populations (human vs. porcine) as well as the influence of underlying disease processes or pathological factors on intestinal mucosal barrier function are suspected in respect that health animals were used in this study.

## Conclusions

In conclusion, the results of our study indicated that damage of intestinal mucosal barrier function with loss of epithelial tight junction leading to bacterial translocation is induced during VV ECMO therapy. The combined use of VV ECMO and CRRT can alleviate levels of intestinal mucosal barrier dysfunction and related bacterial translocation, which may extenuate the ECMO-associated SIRS and raise the effect and safety of VV ECMO.

## Abbreviations

ECMO: Extracorporeal membrane oxygenation; CRRT: Continuous renal replacement therapies; VV ECMO: Venovenous extracorporeal membrane oxygenation; DAO: Diamine oxidase; I-FABP: Fatty acid binding protein; MODS: Multiple organ dysfunction syndrome; ACT: Activated clotting time; CBP: Continuous blood purification; ROS: Reactive oxygen species; iNOS: Induced nitric oxide synthase; NO: Nitric oxide; SIRS: Systemic inflammatory response syndrome.

## Competing interests

The authors declare that they have no competing interests.

## Authors’ contributions

CH, SY and WY are co-first authors and completed most the scientific work and drafted the manuscript. JS, QC performed the model of ECMO. JS completed the statistic work. YH helped the first authors to finish the study. XW, NL, JL helped to conceptualize and design the study and NL was corresponding author for the paper. All authors read and approved the final manuscript.

## Authors’ information

Changsheng He, Shuofei Yang and Wenkui Yu are the co-first authors.
